# The Role of Non-Coding RNAs in the Pathogenesis and Progression of Diabetic Kidney Disease

**DOI:** 10.3390/ijms27052352

**Published:** 2026-03-03

**Authors:** Yinfeng Guo, Yonghao Feng, Henglan Wu, Huanqing Gao

**Affiliations:** 1 Department of Nephrology, Affiliated Hospital of Jiaxing University (The First Hospital of Jiaxing), No.1882, Zhonghuan North Road, Jiaxing 314000, China; xiaoguoyf@163.com (Y.G.); whl525487@sina.com (H.W.); 2 Department of Endocrinology, Jinshan Hospital, Fudan University, Shanghai 201508, China; fengyonghao@fudan.edu.cn; 3 State Key Laboratory of Genetic Engineering, Human Phenome Institute, School of Life Sciences, Fudan University, Shanghai 200438, China

**Keywords:** diabetic kidney disease, non-coding RNAs, microRNAs, long non-coding RNAs, circular RNAs

## Abstract

Diabetic kidney disease (DKD) remains a leading cause of end-stage renal disease worldwide, with current therapies often failing to halt its progression due to an incomplete understanding of intrinsic renal molecular mechanisms. This review highlights the pivotal role of non-coding RNAs (ncRNAs)—including microRNAs (miRNAs), long non-coding RNAs (lncRNAs), and circular RNAs (circRNAs)—as central regulators in the pathogenesis and progression of DKD. We systematically examine how the diabetic milieu dysregulates specific ncRNA profiles in renal cells, driving core pathological processes such as metabolic dysfunction, inflammation, fibrosis, and podocyte injury. Furthermore, we explore the emerging roles of exosomal ncRNAs in intercellular communication and their potential as non-invasive liquid biopsy biomarkers for early diagnosis and disease monitoring. Finally, we discuss the translational prospects of targeting ncRNAs through innovative therapeutic strategies, such as antisense oligonucleotides and miRNA mimics, while addressing the challenges of tissue-specific delivery and clinical implementation. Understanding ncRNA networks offers a refined, systems-level perspective on DKD and opens new avenues for precision diagnostics and targeted interventions aimed at modifying the disease course.

## 1. Introduction

Diabetic kidney disease (DKD) continues to pose a significant global health challenge, being the primary cause of end-stage renal disease (ESRD) and a substantial factor in cardiovascular mortality among individuals with diabetes [1,2,3]. Even though standard-of-care treatments have improved, such as strict glycemic control, blocking the renin–angiotensin–aldosterone system (RAAS), and newer heart- and kidney-protective drugs like SGLT2 inhibitors and GLP-1 receptor agonists, there is still a significant risk of disease progression [4,5]. This clinical inertia highlights a critical deficiency in our present comprehension: the prevailing therapeutic framework predominantly focuses on systemic metabolic and hemodynamic disturbances, yet fails to adequately address the intrinsic, maladaptive molecular reprogramming occurring within the kidney itself. As a result, DKD usually has a steady course, moving from microalbuminuria to obvious proteinuria and a lower glomerular filtration rate (GFR), and finally ending in irreversible kidney failure [6]. This unmet clinical need underscores the necessity to elucidate the exact cellular and molecular mechanisms that inflict damage within the glomerular and tubulointerstitial compartments. It requires a fundamental transformation from perceiving the kidney as a passive target to acknowledging it as an active locus of pathological decision-making.

The pathophysiology of diabetic kidney disease constitutes a complex, multifactorial cascade initiated by the diabetic environment and mediated through intricate intrarenal mechanisms. It is distinguished by a sequence of interconnected pathological features: initial glomerular hyperfiltration and hypertrophy, persistent microinflammation, podocyte damage and loss, and advancing fibrosis of the glomerular and tubulointerstitial regions [7,8]. Conventional pathophysiological models predominantly emphasize linear signaling pathways, in which stimuli like hyperglycemia or transforming growth factor-beta (TGF-β) directly activate transcription factors to facilitate the expression of protein-coding genes associated with inflammation, hypertrophy, and extracellular matrix synthesis [9]. Nonetheless, this linear framework does not accommodate the extraordinary cell-type specificity, temporal dynamics, and regulatory intricacy evident in DKD. It fails to sufficiently elucidate why only a specific subset of patients advances to advanced disease despite analogous metabolic profiles, nor does it encompass the intricate layers of gene regulation that dictate cellular fate decisions between adaptation and maladaptation. This lack of understanding suggests that there is a more complex regulatory layer that interprets long-term metabolic stress and carefully coordinates the pathological response that follows. The rise of non-coding RNAs (ncRNAs) as primary regulators of gene expression presents a significant new perspective for analyzing this complexity, establishing them not only as biomarkers but also as pivotal orchestrators of the pathogenic symphony in diabetic kidney disease.

The conventional protein-centric perspective of the genome has been profoundly transformed by the revelation that the predominant portion of transcriptional output comprises non-coding RNAs (ncRNAs), which are essential for the meticulous regulation of gene expression [10]. These functional RNAs, which do not act as templates for protein synthesis, encompass essential categories such as microRNAs (miRNAs), long non-coding RNAs (lncRNAs), and circular RNAs (circRNAs). miRNAs act as post-transcriptional repressors by directing the RNA-induced silencing complex (RISC) to mRNA targets that are complementary to them. This stops translation or breaks down the transcript [11,12,13]. Long non-coding RNAs (lncRNAs) can be used as scaffolds, decoys, guides, or signals to change the structure of chromatin, control transcription, and change how proteins work [14]. CircRNAs, which have a covalently closed circular structure, often act as “sponges” for competitive endogenous RNA (ceRNA) that trap miRNAs and let their target genes work again [15]. Together, these ncRNAs make up complex, multi-layered regulatory networks that control almost all cellular processes, from development and differentiation to stress responses and pathological remodeling. Within this framework, we intend to methodically investigate their particular role in diabetic kidney disease. This review integrates contemporary evidence to clarify how the diabetic environment disrupts specific ncRNA profiles in renal cells, elucidates the mechanisms by which these dysregulated ncRNAs propel the fundamental pathological processes of diabetic kidney disease—including metabolic dysfunction, inflammation, fibrosis, and podocyte injury—and rigorously assesses their emerging potential as innovative diagnostic biomarkers and therapeutic targets. This viewpoint provides a novel framework for comprehending DKD pathogenesis beyond traditional protein-centric models.

Before discussing specific dysregulated ncRNAs and their disease-module functions in DKD, it is useful to briefly outline the fundamental mechanisms by which different ncRNA classes regulate gene expression, which may help orient readers from diverse disciplinary backgrounds. miRNAs primarily function at the post-transcriptional level by incorporating into the RNA-induced silencing complex (RISC), where they promote target mRNA degradation or translational repression and thereby fine-tune signaling networks involved in fibrosis, inflammation, oxidative stress, and cell survival. In contrast, lncRNAs exhibit broader mechanistic versatility and can act as guides, scaffolds, decoys, or signals in the nucleus and cytoplasm, regulating chromatin organization, transcription factor activity, RNA splicing, mRNA stability, and translation in a context-dependent manner (including cis- and trans-acting effects) [16]. CircRNAs, due to their covalently closed structure and increased stability, are most commonly described as miRNA sponges and protein-interacting regulators, with emerging evidence for additional roles in transcriptional and translational control. In DKD, chronic hyperglycemia, lipotoxicity, oxidative stress, and hemodynamic stress perturb these ncRNA-mediated regulatory layers in podocytes, mesangial cells, tubular epithelial cells, endothelial cells, and infiltrating immune cells, leading to the dysregulation of key pathogenic pathways such as TGF-β/Smad, NF-κB, and PI3K/Akt signaling.

## 2. The Landscape of Dysregulated Non-Coding RNAs in DKD

The diabetic kidney exhibits a significantly altered transcriptional and post-transcriptional landscape, with non-coding RNAs (ncRNAs) serving as critical dysregulated molecular signatures. Making a list of these changes is the first step toward figuring out what they mean for how diseases develop.

### 2.1. MicroRNAs (miRNAs): The Exact Post-Transcriptional Managers

MiRNAs are small (~22 nucleotides) RNAs that have been around for a long time and help control gene expression by binding to complementary sequences in the 3′-untranslated regions (3′UTRs) of target mRNAs. This stops translation or breaks down mRNA. In diabetic kidney disease, specific miRNA profiles have been consistently linked to the progression of the disease [17]. Among the most significantly upregulated miRNAs, miR-21 emerges as a principal pro-fibrotic regulator. It directly affects the tumor suppressor PTEN and the anti-fibrotic Smad7, which increases TGF-β/Smad signaling and encourages the buildup of extracellular matrix (ECM) [18]. MiR-192, which is more active in glomerular mesangial cells and tubular epithelium, makes fibrosis worse by targeting ZEB1/2, which are important blockers of collagen transcription [19]. MiR-377 worsens oxidative stress and fibrosis by stopping antioxidant enzymes like superoxide dismutase (SOD1/2) and the anti-fibrotic protein p21-activated kinase (PAK1) [20]. On the other hand, many protective miRNAs are less active in DKD. The miR-29 family (miR-29a/b/c) protects against fibrosis. When it is lost, it turns on many collagen genes and other ECM components [21]. MiR-25, which is often lower in podocytes, makes its target, the mitochondrial fission regulator Drp1, more active, which causes mitochondrial dysfunction and apoptosis [22]. Members of the let-7 family, which are less active in hyperglycemia, usually stop pathways that cause inflammation and fibrosis [23]. When they are less active, they remove an important brake on the progression of disease.

### 2.2. Long Non-Coding RNAs (lncRNAs): The Conductors of Complex Networks

LncRNAs (>200 nucleotides) are multifunctional regulators that coordinate gene expression through diverse mechanisms, including acting as decoys, scaffolds, guides, and competitive endogenous RNAs (ceRNAs) [24]. In DKD, their relevance lies not only in the effects of individual transcripts, but also in their capacity to integrate hyperglycemia-driven stress signals into coordinated inflammatory, fibrotic, and cell-injury programs.

A recurring theme in DKD is the upregulation of stress-responsive lncRNAs that amplify maladaptive inflammatory and profibrotic signaling. For example, MALAT1 is frequently upregulated under diabetic conditions and has been linked to inflammatory activation and fibroblast-related responses, partly through interactions with miRNA-regulated pathways [25]. Similarly, NEAT1, a key architectural component of paraspeckles, is induced in stress states and can facilitate the expression of pro-inflammatory and pro-fibrotic genes [26,27]. Rather than functioning as isolated downstream markers, these lncRNAs may act as molecular organizers that help stabilize pathogenic transcriptional programs in the diabetic kidney.

Another important mechanism is ceRNA-mediated derepression of pathogenic targets. H19, an imprinted lncRNA dysregulated in DKD, has been reported to sequester miR-29a and let-7 family members, thereby relieving repression of fibrosis- and inflammation-related targets such as TGF-β and COL1A1 [28,29]. Through this mechanism, H19 may contribute to a feed-forward loop that reinforces extracellular matrix accumulation and chronic renal injury.

Not all lncRNAs act uniformly across cell types or disease stages. TUG1 appears to be more context-dependent and has been implicated in the regulation of apoptosis and metabolic homeostasis in podocytes and tubular cells, potentially through chromatin-associated and miRNA-interacting mechanisms [30,31]. This context dependence highlights a broader principle in DKD lncRNA biology: the functional impact of a given lncRNA is shaped by cell type, disease stage, and the surrounding regulatory network.

Collectively, these examples indicate that lncRNAs in DKD are best understood as coordinators of interconnected pathogenic pathways—particularly inflammation, fibrosis, and metabolic/cellular stress responses—rather than as a simple list of independently acting transcripts.

### 2.3. Circular RNAs (circRNAs): The New Stable Regulators

CircRNAs have unique structures in that they are covalently closed loops. This makes them very stable and resistant to degradation by exonucleases. This stability, along with their high expression in specific cell types, makes them good candidates for disease biomarkers and regulators. In DKD, various circRNAs exhibit differential expression [32,33]. For example, circRNA_15698 has been reported to be upregulated and may act as a miRNA “sponge” for miR-185, which usually targets TGF-β1 [32]. This could lead to fibrotic signaling. CircHIPK3 is another abundant circRNA that is involved in cell growth and fibrosis. It can sponge up several miRNAs, such as miR-326 and miR-379, which affect pathways that are important for mesangial cell dysfunction and matrix expansion [34]. Investigations into renal circRNAs are swiftly advancing, focusing on their functions as miRNA reservoirs, protein decoys, or potential templates for translation within the framework of diabetic injury.

### 2.4. Layers of Regulation: Epigenetic Regulation of ncRNA Expression

The expression of ncRNAs is not fixed; rather, it is dynamically regulated by the diabetic milieu, chiefly via epigenetic modifications. DNA methylation at promoter regions is a key switch; hypermethylation can turn off tumor-suppressive miRNAs like miR-200c or miR-29a [35,36], while hypomethylation can turn on profibrotic lncRNAs like H19 [37,38]. Changes to histones are also very important. For instance, high glucose can cause repressive histone marks (like H3K9me3 and H3K27me3) to show up at the promoters of protective miRNAs (like miR-133a) [39]. On the other hand, activating marks (like H3K4me3 and H3K9ac) are more common at the loci of pro-fibrotic lncRNAs like MALAT1 [40]. These epigenetic changes, which are caused by metabolic intermediates (like S-adenosylmethionine and α-ketoglutarate) and oxidative stress, make a “memory” of metabolic damage that keeps the ncRNA landscape off balance, even when glycemic control gets better. This layer of regulation shows that ncRNA dysregulation in DKD is both a result and a cause that keeps the disease going.

**Figure 1:**
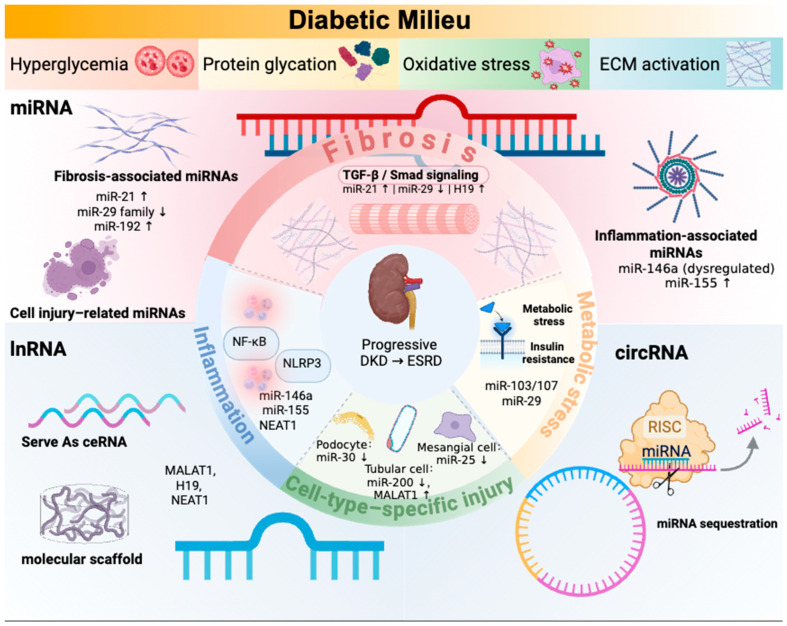
Non-coding RNA-centered regulatory network in diabetic kidney disease (DKD). DKD progression is initiated by stress signals in the diabetic milieu, with dysregulated non-coding RNAs (ncRNAs) acting as central hubs to mediate fibrosis, inflammation, metabolic dysfunction, and cell-type–specific injury. The figure presents a vertical, top-to-bottom regulatory hierarchy: (1) Diabetic Milieu layer includes hyperglycemia, oxidative stress, AGEs, and TGF-β; (2) Core ncRNA layer shows key molecular dysregulation and targets (e.g., miR-21↑ inhibiting SMAD7, MALAT1↑ regulating TGF-β1 as a ceRNA, circ_15698↑ sponging miR-185); (3) Core pathological layer centers on fibrosis, linked to inflammation, metabolic stress, and specific injuries of podocytes, tubular epithelial cells, and mesangial cells; (4) Clinical outcome layer depicts progressive DKD leading to end-stage renal disease (ESRD). Solid arrows indicate activation; T-shaped lines indicate inhibition; ↑/↓ denote upregulation/downregulation in DKD. Created in BioRender. Zhang, H. (2026) https://BioRender.com/kcirp24, accessed on 4 February 2026.

Figure 1 summarizes core regulatory networks of Non-coding RNAs in diabetic nephropathy.

## 3. Functional Mechanisms: The Role of ncRNAs in Core DKD Pathologies

The disordered ncRNA landscape delineated in Part 1 is not a mere epiphenomenon; it represents the operational layer that actively orchestrates the execution of diabetic kidney injury. NcRNAs operate as precise molecular switches and amplifiers by integrating into and frequently dominating established disease pathways. This part breaks down how they work in the main pathophysiological modules of DKD (Table 1).

### 3.1. Module 1: Controlling Insulin Signaling and Metabolic Disturbance

Diabetes causes a basic metabolic problem that the body responds to in a complicated way using ncRNA. This response can either make insulin resistance and bad fuel use in the kidney worse or better.

The miR-29/103/107 Families: These miRNAs are very important for changing how insulin works. MiR-103/107 are increased in insulin-resistant conditions and directly target Caveolin-1, which is an important protein for keeping insulin receptors stable at the plasma membrane [41]. Their overexpression disrupts insulin receptor signaling, leading to renal insulin resistance. On the other hand, the miR-29 family, which is often downregulated, affects how glucose is used in the body. Its targets are PPARδ and GLUT4, and losing it can make renal cells less flexible in their metabolism [42].

LncRNA GAS5 (Growth Arrest-Specific 5): This lncRNA works as a metabolic sensor and stops tumors from growing. Its expression is frequently inhibited in nutrient-rich environments (e.g., hyperglycemia). GAS5 can bind to and block the glucocorticoid receptor (GR) and other transcription factors [43,44]. This changes how gluconeogenic and lipogenic genes are expressed. Its downregulation in DKD may alleviate the suppression of genes that enhance renal gluconeogenesis and metabolic stress, thereby connecting cellular energy status to transcriptional output.

### 3.2. Module 2: Managing Inflammation and the Immune Response

ncRNAs that fine-tune innate immune signaling pathways tightly control the sterile inflammation that is a hallmark of DKD.

MiR-146a: A Regulator of Negative Feedback: When NF-κB is activated by inflammatory signals like AGEs, miR-146a creates an important negative feedback loop [45]. It targets the important signaling adaptors IRAK1 and TRAF6 in the TLR/NF-κB pathway, which lowers the production of pro-inflammatory cytokines like IL-6 and TNF-α [46]. If it does not work correctly, it can break this feedback loop, which can cause inflammation to last.

MiR-155 and LncRNA NEAT1 are molecules that activate the inflammasome. MiR-155 is a strong pro-inflammatory miRNA that targets SOCS1, which stops inflammasome signaling, to help NLRP3 inflammasome assembly. Likewise, lncRNA NEAT1 is elevated in diabetic kidneys and is crucial for the development of nuclear paraspeckles. It promotes NLRP3 inflammasome activation by facilitating the transcriptional upregulation of its components and interacting with inflammasome-related proteins, leading to caspase-1 activation and IL-1β/IL-18 maturation, which drive pyroptosis and amplify tissue inflammation [47,48].

### 3.3. Module 3: Taking Control of the Fibrotic Cascade

Fibrosis is the ultimate shared mechanism leading to renal failure, with ncRNAs constituting a principal regulatory network governing this process.

The miR-29 Family: The Main Anti-Fibrotic Hub: The miR-29 family is probably the strongest natural blocker of fibrosis. It directly attacks the mRNAs of more than a dozen ECM parts, such as COL1A1, COL3A1, COL4A1, COL5A1, FBN1, ELN, and pro-fibrotic regulators like TGF-β1, CTGF, and IGF-1 [49,50,51]. TGF-β1 and high blood sugar levels consistently lower it in DKD, which removes a strong brake on ECM production and causes fibrosis.

MiR-21: The Pro-Fibrotic Executor: In DKD fibrosis, MiR-21 is one of the miRNAs that is most upregulated. It promotes fibrosis mainly by inhibiting SMAD7, which is an important intracellular blocker of the TGF-β/SMAD signaling pathway. miR-21 enhances and maintains TGF-β signaling by inhibiting SMAD7 [18,52]. It also affects PTEN, which activates AKT and helps fibrogenic cells survive and grow [53,54].

LncRNA H19: The Fibrosis Network Enhancer: H19 is a key part of a competitive endogenous RNA (ceRNA) network. There are a lot of places where miR-29a and let-7 can bind to it [55]. H19 acts like a molecular “sponge” to hold onto these protective miRNAs so that they cannot interact with their target mRNAs. This sequestration leads to the derepression of a large number of fibrotic genes, such as collagens and TGF-β pathway components, which greatly increases the fibrotic signal.

### 3.4. Module 4: Mediating Cell-Type-Specific Injury

Specialized ncRNA programs control the different weaknesses of different types of kidney cells.

Podocytes and maintenance of the glomerular filtration barrier

Family of MiR-30: This family is very important for keeping podocytes healthy and differentiated. It stops Snail1, which is a master regulator of epithelial-to-mesenchymal transition (EMT), and controls genes related to autophagy, such as Beclin-1 [56,57,58]. Its downregulation in DKD results in podocyte dedifferentiation, compromised autophagy, and heightened vulnerability to apoptosis.

LncRNA TUG1: Shows different roles depending on the situation. In certain studies, the downregulation of TUG1 correlates with high glucose-induced podocyte apoptosis, possibly via the dysregulation of PGC-1α-mediated mitochondrial biogenesis [59,60]. It might serve as a framework for chromatin-modifying complexes that influence survival genes.

Tubular Epithelial Cells: The First Line of Defense Against Injury

MiR-200 Family: Important for EMT and maintaining tubular epithelial homeostasis. MiR-200b/c targets ZEB1/ZEB2, which are transcriptional repressors of E-cadherin. When tubular cells are stressed by diabetes, they lose miR-200, which speeds up EMT, a key step in tubulointerstitial fibrosis [61,62].

LncRNA MALAT1: When glucose levels are high, MALAT1 is turned on in tubular cells. It activates NF-κB, which causes inflammation, and it acts as a ceRNA for miRNAs like miR-145, which makes TGF-β1 and fibronectin more active. This causes tubular injury and interstitial fibrosis [63,64,65].

Mesangial Cells: Builders of the Glomerular Matrix

MiR-25: Controls the balance of mesangial cells. In a diabetic state, modified miR-25 expression impacts its targets, such as Drp1 (which controls mitochondrial fission and apoptosis) and NOX4 (a primary source of renal reactive oxygen species). This affects the growth of mesangial cells, oxidative stress, and, in the end, the expansion of the pathological matrix [66,67].

While these studies collectively support important roles for ncRNAs in core DKD pathologies, most available evidence has been generated from bulk tissue analyses or reductionist cell/animal models, which may not fully capture the marked cellular heterogeneity of the diabetic kidney. In this context, single-cell RNA sequencing (scRNA-seq) and single-nucleus RNA sequencing (snRNA-seq) offer a valuable framework to refine ncRNA-associated mechanisms at cell-type resolution. These approaches can help determine whether dysregulated ncRNA signatures arise predominantly in podocytes, proximal/distal tubular epithelial cells, mesangial cells, glomerular endothelial cells, or infiltrating immune populations, and in which pathological contexts they are most relevant (e.g., podocyte injury, tubular metabolic stress, mesangial matrix expansion, endothelial dysfunction, or inflammatory activation). Such resolution may also improve interpretation of exosomal ncRNA signals by helping infer their likely cellular origin and biological context [68]. However, because standard single-cell workflows may incompletely capture some ncRNA species—particularly low-abundance lncRNAs and many circRNAs—future studies will likely require integration with complementary approaches, including snRNA-seq, small RNA sequencing, spatial transcriptomics, and targeted validation, to achieve a more comprehensive map of ncRNA regulation in DKD.

## 4. The Communicative Aspect: Exosomal ncRNAs in DKD

The pathophysiology of DKD involves more than just cells not working properly on their own; it also includes a complex system of communication between cells. Exosomes, which are small extracellular vesicles (30–150 nm), are at the heart of this conversation [69]. They are important for the targeted delivery of non-coding RNAs, which helps different types of renal cells and compartments respond to disease in a coordinated way.

### 4.1. Exosomes as ncRNA Delivery Vehicles: Biogenesis and Reprogramming in Diabetes

Endosomal membranes bud inward to form exosomes. These vesicles are called intraluminal vesicles and are found inside multivesicular bodies (MVBs). They then fuse with the plasma membrane to be released. Their cargo, which includes proteins, lipids, and nucleic acids, is selectively packaged to reflect the state of the parent cell. In the context of diabetes, this packaging process becomes unregulated [70,71]. Hyperglycemia, oxidative stress, and inflammatory cytokines (e.g., TGF-β) modify the molecular mechanisms governing exosome biogenesis and cargo sorting, resulting in the selective enrichment of specific pathogenic non-coding RNAs [72,73]. For example, enzymes like nSMase2, which controls the formation of ceramide-dependent exosomes, are upregulated when someone has diabetes, which makes vesicles secrete more [74]. At the same time, RNA-binding proteins (like hnRNPA2B1 and Y-box protein 1) that find certain patterns in ncRNAs and help them get into exosomes are changed. This creates an exosomal “signalosome” that carries a unique, disease-promoting signature from stressed renal cells into the blood and the area around them [75].

### 4.2. Pathogenic Messengers: Coordinating a Paracrine Disease Network

Exosomes serve as vehicles for transmitting harmful signals. Stressed or damaged renal cells, including podocytes, tubular epithelial cells, and activated macrophages, secrete exosomes containing pathogenic miRNAs [76,77].

MiR-21-Enriched Exosomes: Tubular cells that are exposed to a lot of glucose release exosomes that have a lot of miR-21 in them. When resident fibroblasts or other tubular cells take up this exosomal miR-21, it stops PTEN and SMAD7 in the cells that take it up, which turns on the AKT and TGF-β/SMAD pathways [78]. This causes fibroblasts to become active, divide, and make collagen, which directly leads to tubulointerstitial fibrosis.

Exosomes with a lot of miR-192: Glomerular mesangial cells release exosomes that have a lot of miR-192 in them. When podocytes take in these exosomes, the miR-192 they contain lowers the levels of ZEB1/2, which makes collagen genes more active and makes glomerular sclerosis worse. This is a direct way for glomerular cells to talk to each other, which makes the injury worse [79,80,81].

This transfer through exosomes makes a loop that amplifies both paracrine and endocrine signals. A small group of damaged cells can use exosomes to reprogram a much larger group of nearby cells, putting them all in a pro-fibrotic and pro-inflammatory state, which speeds up the progression of the disease in a coordinated way.

### 4.3. Protective Signals: Using Exosomes for Treatment

On the other hand, the exosomal delivery system can be used for medical purposes. Exosomes derived from mesenchymal stem cells (MSCs) have surfaced as a promising cell-free regenerative therapy. These exosomes come with a lot of protective ncRNAs built in.

MiR-let-7c: MSC-exosomal miR-let-7c can be transported to renal tubular cells, where it specifically targets TGFBR1 and HMGA2, which are important for fibrosis and inflammation. This stops TGF-β signaling and EMT [82].

MiR-486: This miRNA, abundant in MSC exosomes, can impede SMAD2 phosphorylation and downregulate NOX4, thereby diminishing oxidative stress and apoptosis in recipient podocytes and tubular cells [83].

The therapeutic efficacy of these exosomes is attributed to their biological membrane, which provides immune privilege and natural tissue tropism, facilitating the targeted delivery of their protective ncRNA cargo to injured sites, thereby promoting repair and reducing damage without the risks inherent in whole-cell transplantation.

### 4.4. Possibility of “Liquid Biopsy”: A Glimpse into the Diabetic Kidney

The stability of exosomes and their ncRNA cargo in biofluids (plasma, urine) offers a revolutionary opportunity for non-invasive “liquid biopsy.” Exosomes that are circulating act like pictures of the cells they came from. In DKD: Urinary exosomal miRNAs (e.g., elevated miR-21, miR-29c) directly indicate the stress state of renal parenchymal cells and correlate with the severity of tubulointerstitial fibrosis and decreasing eGFR [84,85].

Plasma exosomal lncRNAs (e.g., MALAT1, H19) may elucidate systemic endothelial dysfunction and inflammatory conditions linked to the progression of DKD [86,87].

Examining exosomal ncRNA profiles is much better than looking at all of the RNA in plasma or urine because it keeps the cargo from breaking down and gives information that is specific to each cell type. Future diagnostic platforms seek to delineate multi-ncRNA signatures from exosomes capable of differentiating early stages of diabetic kidney disease, forecasting rapid disease progression, and facilitating real-time monitoring of therapeutic responses, thereby inaugurating an era of precision nephrology.

**Figure 2:**
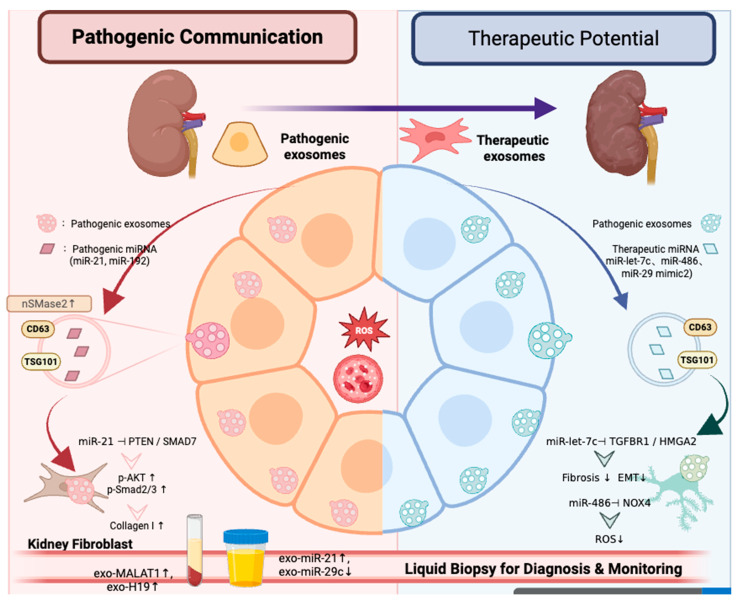
Exosome-mediated ncRNA communication and translational implications in DKD. Left (light red theme): Injured tubular epithelial cells release pathogenic exosomes (marked by CD63/TSG101) containing miR-21 and miR-192. Uptaken by renal fibroblasts, these miRNAs inhibit PTEN/SMAD7, promoting collagen expression and fibrosis. Right (light blue theme): Mesenchymal stem cell (MSC)-derived exosomes deliver miR-let-7c, miR-486, and miR-29 mimic to injured podocytes, targeting TGFBR1 and NOX4 to reduce fibrosis and apoptosis. Bottom: Urine shows exo-miR-21↑ and exo-miR-29c↓, while plasma exhibits exo-MALAT1↑ and exo-H19↑, which serve as non-invasive diagnostic and monitoring biomarkers for DKD. Created in BioRender. Zhang, H. (2026) https://BioRender.com/qo2da8w, accessed on 4 February 2026.

Figure 2 summarizes Exosome-mediated intercellular communication mechanism of ncRNA.

## 5. Translational Perspectives: From Biomarkers to Therapeutic Targets

The significant dysregulation of ncRNAs in DKD establishes them as both mechanistic linchpins and prime candidates for clinical translation. This section examines the dual potential of ncRNAs as next-generation diagnostic instruments and actionable therapeutic targets, while rigorously evaluating the significant challenges encountered in the transition from laboratory to clinical application.

### 5.1. New Biomarker Potential: Moving Toward Precision Monitoring

The stability of ncRNAs, especially in biofluids, presents a promising opportunity for non-invasive “liquid biopsies” to revolutionize the management of DKD.

Circulating miRNAs: Distinct serum/plasma miRNA signatures exhibit a robust correlation with the presence and progression of DKD. High levels of miR-21 are strongly linked to more severe tubulointerstitial fibrosis and a lower eGFR, making it a possible prognostic marker [88]. On the other hand, lower levels of miR-126, a type of miRNA that is found mostly in endothelial cells, are linked to microalbuminuria and endothelial dysfunction, which are signs of early damage to blood vessels [89].

Urinary ncRNAs provide even more direct information about the kidneys. The urinary excretion of lncRNA TUG1 is associated with podocyte injury, whereas altered levels of miR-29c and miR-200b in urine sediment indicate active fibrotic and epithelial–mesenchymal transition (EMT) processes in the tubulointerstitium, potentially surpassing albuminuria in predicting histopathological changes [60,90].

### 5.2. Therapeutic Targeting Strategies: Silencing and Replenishing the Regulatory Network

Many ncRNAs are directly druggable because they are involved in DKD. Strategies can be divided into two groups: those that stop harmful molecules and those that restore protective ones.

Inhibiting Pathogenic ncRNAs: This method uses drugs made of nucleic acids to silence ncRNAs that are overexpressed.

Antisense Oligonucleotides (ASOs) and Tiny LNAs are chemically altered single-stranded RNAs that are made to bind to complementary sequences in target miRNAs or lncRNAs. This causes RNase H-mediated degradation or steric blockade. In preclinical studies, subcutaneous delivery of a miR-21 ASO (e.g., Lademirsen) markedly reduced renal fibrosis and inflammation in diabetic mouse models. Likewise, ASOs directed at lncRNA MALAT1 have demonstrated effectiveness in mitigating renal injury [91,92].

Small Interfering RNAs (siRNAs): Traditionally utilized for mRNAs, sophisticated siRNA configurations can also specifically target and degrade certain lncRNA transcripts. FDA-approved drugs like Patisiran show that they are a good platform because they are very specific and powerful [93,94].

miRNA Mimics (Agomirs): These are RNA molecules that are double-stranded and look and work like natural protective miRNAs. The systemic administration of a miR-29b agomir in diabetic rodents effectively diminished renal collagen accumulation and arrested the progression of fibrosis. Let-7c agomirs have exhibited comparable potential in mitigating tubular injury and inflammation [95,96].

Lipid Nanoparticles (LNPs): The technology that makes COVID-19 mRNA vaccines possible, LNPs can be designed to hold and protect ncRNA drugs, which makes it easier for cells to take them in. Changing the surface properties of LNPs (PEGylation, targeting ligands) can help them spread throughout the body better [97,98]. Nanomedicine-based targeted delivery strategies are increasingly relevant to ncRNA therapeutics for DKD, as they may address a major translational bottleneck: efficient kidney and renal cell-specific delivery with limited systemic off-target exposure. Although recent discussions [99] have focused mainly on acute kidney injury, the underlying design principles are highly applicable to DKD, including optimization of nanoparticle size, surface charge, and composition to improve renal biodistribution, cargo protection, cellular uptake, and controlled release.

Exosome Mimetics: Creating synthetic vesicles or using native exosomes from certain cell types (like mesenchymal stem cells) as targeted delivery vehicles for ncRNA drugs is a new and exciting idea.

### 5.3. Problems and Next Steps

Even though there is exciting preclinical proof-of-concept, there are still some major problems that need to be solved:

Specificity for Tissues and Cells: It is very important to avoid unintended effects in other organs. The most difficult part is making delivery systems that really only affect the renal parenchyma.

Off-Target Effects and Long-Term Safety: Nucleic acid therapeutics may disrupt the expression of unintended genes through sequence homology or immune activation (e.g., via Toll-like receptors). It is important to do thorough off-target screening and long-term toxicology studies.

Delivery Efficiency: Getting enough of the drug into the right type of renal cell (podocytes or fibroblasts) after it has been given systemically is still a big problem in pharmacology.

Therapy in combination Reason: Rational polytherapy is probably the way of the future. An ASO targeting miR-21 could be effectively synergized with an SGLT2 inhibitor, which enhances the metabolic environment. A miR-29 mimic could also work with a GLP-1 receptor agonist to fight fibrosis from different angles. Defining these kinds of synergistic combinations will be very important for getting the most out of them and making them last longer.

Biomarker-Driven Trials: Future clinical trials ought to implement a precision medicine strategy, enrolling patients according to their ncRNA biomarker profile (e.g., high miR-21 expressors) to ascertain the population most likely to benefit from targeted therapy, thereby enhancing trial success rates.

## 6. Conclusions

In summary, the transition of ncRNA biology into clinical applications for DKD involves progression from correlation to causation, culminating in intervention. Although the path is complicated, the combination of advanced nucleic acid chemistry, new delivery platforms, and a deep understanding of how things work is opening up a new frontier in the treatment of this debilitating disease, bringing us closer to real disease modification. Non-coding RNAs have arisen not as peripheral components but as central orchestrators of DKD pathogenesis. Their research offers more sophisticated, network-oriented comprehension of disease progression and reveals unparalleled prospects for precision diagnostics and targeted therapeutics, ultimately instilling hope for modifying the unyielding trajectory of diabetic kidney disease.

## Figures and Tables

**Table 1:** Representative ncRNAs implicated in DKD across the three major classes.

ncRNA Class	Representative ncRNA (Examples)	Reported Change in DKD	Major Renal Cell Types/Compartments	Key Targets or Pathways (Representative)	Main DKD-Related Functions/Phenotypes	Translational Relevance (Biomarker/Therapeutic)
miRNA	miR-21	Usually ↑	Tubular epithelial cells, mesangial cells, interstitium	TGF-β/Smad-related signaling, PTEN/AKT, pro-fibrotic/inflammatory pathways	Promotes fibrosis, inflammation, and renal injury progression	Candidate therapeutic target (anti-miR strategy); also biomarker candidate in biofluids/exosomes
miRNA	miR-29 family (e.g., miR-29a/b/c)	Often ↓ (context-dependent)	Mesangial cells, fibroblast-related pathways, tubulointerstitium	ECM/collagen/fibrotic gene networks	Generally antifibrotic; loss associated with ECM accumulation and fibrosis	Potential anti-fibrotic therapeutic axis; biomarker relevance under investigation
miRNA	miR-192	Context-/stage-dependent (often altered in DKD)	Mesangial cells, glomerular compartment	TGF-β-associated regulatory network, fibrosis-related signaling	Linked to glomerular fibrosis and disease progression (direction may vary by model/stage)	Primarily mechanistic and biomarker interest; therapeutic role requires context stratification
miRNA	miR-146a	Often ↓ in injury/inflammation contexts	Podocytes, immune-related renal microenvironment	NF-κB/inflammatory signaling pathways	Anti-inflammatory and renoprotective effects in several models	Potential protective miRNA replacement strategy; biomarker interest
miRNA	miR-200 family	Altered (context-dependent)	Tubular epithelial cells, mesangial cells	EMT/fibrosis-related pathways (e.g., ZEB/TGF-β axis)	Involved in epithelial injury/fibrotic remodeling	Mechanistic significance; biomarker/therapy potential still being refined
lncRNA	MALAT1	Usually ↑	Podocytes, tubular cells, endothelial/mesangial contexts	Inflammatory and fibrotic signaling; ceRNA-like regulation	Promotes inflammation, oxidative stress, fibrosis, and cell injury in hyperglycemic conditions	Biomarker and target candidate; requires tissue/cell-type-specific validation
lncRNA	PVT1	Usually ↑	Mesangial cells, glomerular compartments	ECM/fibrosis-related signaling networks	Associated with ECM accumulation and fibrotic remodeling	Mechanistic and biomarker interest; therapeutic translation remains early
lncRNA	TUG1	Often ↓ in podocyte injury contexts	Podocytes (especially mitochondrial stress contexts)	Mitochondrial bioenergetics/PGC-1α-related pathways (reported)	Generally protective; supports mitochondrial function and podocyte survival	Potential renoprotective target axis; delivery feasibility remains a challenge
lncRNA	NEAT1	Usually ↑	Tubular cells, mesangial cells, inflammatory microenvironment	ceRNA-associated inflammatory/fibrotic signaling	Promotes inflammation, apoptosis, and fibrosis-related responses	Candidate biomarker/target; mechanism depends on interacting miRNA/mRNA axes
lncRNA	MEG3	Altered (often ↑ in DKD injury models)	Podocytes, mesangial/tubular contexts	Apoptosis-, fibrosis-, and inflammation-related signaling	Associated with cell injury, fibrosis, and progression-related pathways	Mechanistic candidate; translational evidence still limited
circRNA	circHIPK3	Usually ↑ (reported in several DKD models)	Tubular cells, mesangial cells, podocyte-related contexts	Frequently reported as miRNA sponge; downstream injury/fibrosis pathways	Promotes inflammation, fibrosis, oxidative stress, and/or apoptosis (model-dependent)	Strong biomarker interest (stability); therapeutic targeting still early
circRNA	circRNA_15698 (example from DKD models)	↑ (reported in experimental DKD)	Mesangial cells/glomerular context	Fibrosis-associated axes (reported miRNA-mediated regulation)	Promotes ECM accumulation and fibrotic responses	Mechanistic interest; translational data limited
circRNA	Exosome-associated circRNAs (multiple candidates)	Altered in urine/plasma exosomes	Biofluids/extracellular vesicles	Intercellular signaling via miRNA/protein interaction networks	May reflect renal injury state and disease progression	High biomarker potential (non-invasive sampling); requires standardized isolation/normalization

## Data Availability

No new data were created or analyzed in this study.

## References

[1] Li J., Guo K., Qiu J., Xue S., Pi L., Li X., Huang G., Xie Z., Zhou Z. (2025). Epidemiological status, development trends, and risk factors of disability-adjusted life years due to diabetic kidney disease: A systematic analysis of Global Burden of Disease Study 2021. Chin. Med. J..

[2] Liu W.-Y., Chen W.-Y., Zhang J.-H., Targher G., Byrne C.D., Misra A., Lonardo A., Zheng M.-H., Sun D.-Q. (2025). The global burden of diabetes-related chronic kidney disease from 1990 to 2021, with projections to 2036. Metab. Target Organ Damage.

[3] Lonardo A. (2024). Association of NAFLD/NASH, and MAFLD/MASLD with chronic kidney disease: An updated narrative. Metab. Target Organ Damage.

[4] Bae J.H. (2025). SGLT2 Inhibitors and GLP-1 Receptor Agonists in Diabetic Kidney Disease: Evolving Evidence and Clinical Application. Diabetes Metab. J..

[5] Zhang B., Deng L. (2025). Application of SGLT-2 inhibitors in non-diabetic CKD: Mechanisms, efficacy, and safety. Front. Med..

[6] Scilletta S., Di Marco M., Miano N., Filippello A., Di Mauro S., Scamporrino A., Musmeci M., Coppolino G., Di Giacomo Barbagallo F., Bosco G. (2023). Update on Diabetic Kidney Disease (DKD): Focus on Non-Albuminuric DKD and Cardiovascular Risk. Biomolecules.

[7] Lu C.C., Wang G.H., Lu J., Chen P.P., Zhang Y., Hu Z.B., Ma K.L. (2019). Role of Podocyte Injury in Glomerulosclerosis. Adv. Exp. Med. Biol..

[8] Sinha S.K., Nicholas S.B. (2023). Pathomechanisms of Diabetic Kidney Disease. J. Clin. Med..

[9] Tie Y., Tang F., Peng D., Zhang Y., Shi H. (2022). TGF-beta signal transduction: Biology, function and therapy for diseases. Mol. Biomed..

[10] Lozano-Villada S., Puthanveettil S.V. (2025). Noncoding RNAs orchestrating the central dogma. J. Biol. Chem..

[11] Kaikkonen M.U., Lam M.T., Glass C.K. (2011). Non-coding RNAs as regulators of gene expression and epigenetics. Cardiovasc. Res..

[12] Wilczynska A., Bushell M. (2015). The complexity of miRNA-mediated repression. Cell Death Differ..

[13] Bartel D.P. (2004). MicroRNAs: Genomics, biogenesis, mechanism, and function. Cell.

[14] Chodurska B., Kunej T. (2025). Long non-coding RNAs in humans: Classification, genomic organization and function. Noncoding RNA Res..

[15] Li J., Han Y., Wang S., Wu X., Cao J., Sun T. (2023). Circular RNAs: Biogenesis, Biological Functions, and Roles in Myocardial Infarction. Int. J. Mol. Sci..

[16] Statello L., Guo C.J., Chen L.L., Huarte M. (2021). Gene regulation by long non-coding RNAs and its biological functions. Nat. Rev. Mol. Cell Biol..

[17] Rodzon-Norwicz M., Kogut P., Sowa-Kucma M., Gala-Bladzinska A. (2025). What a Modern Physician Should Know About microRNAs in the Diagnosis and Treatment of Diabetic Kidney Disease. Int. J. Mol. Sci..

[18] McClelland A.D., Herman-Edelstein M., Komers R., Jha J.C., Winbanks C.E., Hagiwara S., Gregorevic P., Kantharidis P., Cooper M.E. (2015). miR-21 promotes renal fibrosis in diabetic nephropathy by targeting PTEN and SMAD7. Clin. Sci..

[19] Putta S., Lanting L., Sun G., Lawson G., Kato M., Natarajan R. (2012). Inhibiting microRNA-192 ameliorates renal fibrosis in diabetic nephropathy. J. Am. Soc. Nephrol..

[20] Wang Q., Wang Y., Minto A.W., Wang J., Shi Q., Li X., Quigg R.J. (2008). MicroRNA-377 is up-regulated and can lead to increased fibronectin production in diabetic nephropathy. FASEB J..

[21] Dalgaard L.T., Sorensen A.E., Hardikar A.A., Joglekar M.V. (2022). The microRNA-29 family: Role in metabolism and metabolic disease. Am. J. Physiol. Cell Physiol..

[22] Liu F., Chen J., Luo C., Meng X. (2022). Pathogenic Role of MicroRNA Dysregulation in Podocytopathies. Front. Physiol..

[23] Brennan E., Wang B., McClelland A., Mohan M., Marai M., Beuscart O., Derouiche S., Gray S., Pickering R., Tikellis C. (2017). Protective Effect of let-7 miRNA Family in Regulating Inflammation in Diabetes-Associated Atherosclerosis. Diabetes.

[24] Mattick J.S., Amaral P.P., Carninci P., Carpenter S., Chang H.Y., Chen L.L., Chen R., Dean C., Dinger M.E., Fitzgerald K.A. (2023). Long non-coding RNAs: Definitions, functions, challenges and recommendations. Nat. Rev. Mol. Cell Biol..

[25] Abdulle L.E., Hao J.L., Pant O.P., Liu X.F., Zhou D.D., Gao Y., Suwal A., Lu C.W. (2019). MALAT1 as a Diagnostic and Therapeutic Target in Diabetes-Related Complications: A Promising Long-Noncoding RNA. Int. J. Med. Sci..

[26] Ghafouri-Fard S., Taheri M. (2019). Nuclear Enriched Abundant Transcript 1 (NEAT1): A long non-coding RNA with diverse functions in tumorigenesis. Biomed. Pharmacother..

[27] Pan Y., Wang T., Zhao Z., Wei W., Yang X., Wang X., Xin W. (2022). Novel Insights into the Emerging Role of Neat1 and Its Effects Downstream in the Regulation of Inflammation. J. Inflamm. Res..

[28] Wu Q., Huang F. (2023). LncRNA H19: A novel player in the regulation of diabetic kidney disease. Front. Endocrinol..

[29] Xie H., Xue J.D., Chao F., Jin Y.F., Fu Q. (2016). Long non-coding RNA-H19 antagonism protects against renal fibrosis. Oncotarget.

[30] Ghaforui-Fard S., Vafaee R., Taheri M. (2019). Taurine-upregulated gene 1: A functional long noncoding RNA in tumorigenesis. J. Cell. Physiol..

[31] Guo S., Zhang L., Zhang Y., Wu Z., He D., Li X., Wang Z. (2019). Long non-coding RNA TUG1 enhances chemosensitivity in non-small cell lung cancer by impairing microRNA-221-dependent PTEN inhibition. Aging.

[32] Ma Y., Zheng L., Gao Y., Zhang W., Zhang Q., Xu Y. (2021). A Comprehensive Overview of circRNAs: Emerging Biomarkers and Potential Therapeutics in Gynecological Cancers. Front. Cell Dev. Biol..

[33] Chen X.T., Li Z.W., Zhao X., Li M.L., Hou P.F., Chu S.F., Zheng J.N., Bai J. (2021). Role of Circular RNA in Kidney-Related Diseases. Front. Pharmacol..

[34] Zheng Q., Bao C., Guo W., Li S., Chen J., Chen B., Luo Y., Lyu D., Li Y., Shi G. (2016). Circular RNA profiling reveals an abundant circHIPK3 that regulates cell growth by sponging multiple miRNAs. Nat. Commun..

[35] Sanchez-Ceinos J., Hussain S., Khan A.W., Zhang L., Almahmeed W., Pernow J., Cosentino F. (2024). Repressive H3K27me3 drives hyperglycemia-induced oxidative and inflammatory transcriptional programs in human endothelium. Cardiovasc. Diabetol..

[36] Vrba L., Jensen T.J., Garbe J.C., Heimark R.L., Cress A.E., Dickinson S., Stampfer M.R., Futscher B.W. (2010). Role for DNA methylation in the regulation of miR-200c and miR-141 expression in normal and cancer cells. PLoS ONE.

[37] Natarajan R. (2021). Epigenetic Mechanisms in Diabetic Vascular Complications and Metabolic Memory: The 2020 Edwin Bierman Award Lecture. Diabetes.

[38] Wang T., Cheng M., Jin J., Bai Y., Zhang D., Zhang S., Xu J. (2024). Hypomethylation of the LncRNA H19 promoter accelerates osteogenic differentiation of vascular smooth muscle cells by activating the Erk1/2 pathways. J. Int. Med. Res..

[39] Saviana M., Le P., Micalo L., Del Valle-Morales D., Romano G., Acunzo M., Li H., Nana-Sinkam P. (2023). Crosstalk between miRNAs and DNA Methylation in Cancer. Genes.

[40] Arunkumar G. (2024). LncRNAs: The good, the bad, and the unknown. Biochem. Cell Biol..

[41] Trajkovski M., Hausser J., Soutschek J., Bhat B., Akin A., Zavolan M., Heim M.H., Stoffel M. (2011). MicroRNAs 103 and 107 regulate insulin sensitivity. Nature.

[42] Zhou Y., Gu P., Shi W., Li J., Hao Q., Cao X., Lu Q., Zeng Y. (2016). MicroRNA-29a induces insulin resistance by targeting PPARdelta in skeletal muscle cells. Int. J. Mol. Med..

[43] Zhou Y., Chen B. (2020). GAS5-mediated regulation of cell signaling. Mol. Med. Rep..

[44] Pickard M.R., Williams G.T. (2015). Molecular and Cellular Mechanisms of Action of Tumour Suppressor GAS5 LncRNA. Genes.

[45] Saba R., Sorensen D.L., Booth S.A. (2014). MicroRNA-146a: A Dominant, Negative Regulator of the Innate Immune Response. Front. Immunol..

[46] Han R., Gao J., Wang L., Hao P., Chen X., Wang Y., Jiang Z., Jiang L., Wang T., Zhu L. (2023). MicroRNA-146a negatively regulates inflammation via the IRAK1/TRAF6/NF-kappaB signaling pathway in dry eye. Sci. Rep..

[47] Zhang P., Cao L., Zhou R., Yang X., Wu M. (2019). The lncRNA Neat1 promotes activation of inflammasomes in macrophages. Nat. Commun..

[48] Tastan B., Cotuk A., Arioz B.I., Karacicek B., Ture B., Tastan N., Genc S. (2025). Long non-coding RNA NEAT1 modulates microglial NLRP3 inflammasome activation. J. Neuroimmunol..

[49] Qin W., Chung A.C., Huang X.R., Meng X.M., Hui D.S., Yu C.M., Sung J.J., Lan H.Y. (2011). TGF-beta/Smad3 signaling promotes renal fibrosis by inhibiting miR-29. J. Am. Soc. Nephrol..

[50] Cushing L., Kuang P.P., Qian J., Shao F., Wu J., Little F., Thannickal V.J., Cardoso W.V., Lu J. (2011). miR-29 is a major regulator of genes associated with pulmonary fibrosis. Am. J. Respir. Cell Mol. Biol..

[51] Jensen D.M., Han P., Mangala L.S., Lopez-Berestein G., Sood A.K., Liu J., Kriegel A.J., Usa K., Widlansky M.E., Liang M. (2022). Broad-acting therapeutic effects of miR-29b-chitosan on hypertension and diabetic complications. Mol. Ther..

[52] Zhong X., Chung A.C., Chen H.Y., Meng X.M., Lan H.Y. (2011). Smad3-mediated upregulation of miR-21 promotes renal fibrosis. J. Am. Soc. Nephrol..

[53] Song X., Liu F., Chen M., Zhu M., Zheng H., Wang W., Chen D., Li M., Chen S. (2024). MiR-21 regulates skeletal muscle atrophy and fibrosis by targeting TGF-beta/SMAD7-SMAD2/3 signaling pathway. Heliyon.

[54] Liu S., Wu W., Liao J., Tang F., Gao G., Peng J., Fu X., Zhan Y., Chen Z., Xu W. (2022). MicroRNA-21: A Critical Pathogenic Factor of Diabetic Nephropathy. Front. Endocrinol..

[55] Kallen A.N., Zhou X.B., Xu J., Qiao C., Ma J., Yan L., Lu L., Liu C., Yi J.S., Zhang H. (2013). The imprinted H19 lncRNA antagonizes let-7 microRNAs. Mol. Cell.

[56] Peng R., Zhou L., Zhou Y., Zhao Y., Li Q., Ni D., Hu Y., Long Y., Liu J., Lyu Z. (2015). MiR-30a Inhibits the Epithelial--Mesenchymal Transition of Podocytes through Downregulation of NFATc3. Int. J. Mol. Sci..

[57] Wu J., Zheng C., Fan Y., Zeng C., Chen Z., Qin W., Zhang C., Zhang W., Wang X., Zhu X. (2014). Downregulation of microRNA-30 facilitates podocyte injury and is prevented by glucocorticoids. J. Am. Soc. Nephrol..

[58] Xiong Y., Wang Y., Wang L., Huang Y., Xu Y., Xu L., Guo Y., Lu J., Li X., Zhu M. (2018). MicroRNA-30b targets Snail to impede epithelial-mesenchymal transition in pancreatic cancer stem cells. J. Cancer.

[59] Lei M., Ke G., Wang Y., Luo D., Hu Y. (2022). Long non-coding RNA TUG1 sponges microRNA-9 to protect podocytes from high glucose-induced apoptosis and mitochondrial dysfunction via SIRT1 upregulation. Exp. Ther. Med..

[60] Chen T., Lu J., Fan Q. (2025). lncRNA TUG1 and kidney diseases. BMC Nephrol..

[61] Korpal M., Lee E.S., Hu G., Kang Y. (2008). The miR-200 family inhibits epithelial-mesenchymal transition and cancer cell migration by direct targeting of E-cadherin transcriptional repressors ZEB1 and ZEB2. J. Biol. Chem..

[62] Brabletz S., Brabletz T. (2010). The ZEB/miR-200 feedback loop—A motor of cellular plasticity in development and cancer?. EMBO Rep..

[63] Ran L., Pan W., Feng J., Tang L. (2025). Long non-coding RNA MALAT1: A crucial factor in fibrotic diseases. Mol. Ther. Nucleic Acids.

[64] Yang Z., Song D., Wang Y., Tang L. (2022). lncRNA MALAT1 Promotes Diabetic Nephropathy Progression via miR-15b-5p/TLR4 Signaling Axis. J. Immunol. Res..

[65] Liu B., Qiang L., Wang G.D., Duan Q., Liu J. (2019). LncRNA MALAT1 facilities high glucose induced endothelial to mesenchymal transition and fibrosis via targeting miR-145/ZEB2 axis. Eur. Rev. Med. Pharmacol. Sci..

[66] Tang J., Yao D., Yan H., Chen X., Wang L., Zhan H. (2019). The Role of MicroRNAs in the Pathogenesis of Diabetic Nephropathy. Int. J. Endocrinol..

[67] Oh H.J., Kato M., Deshpande S., Zhang E., Das S., Lanting L., Wang M., Natarajan R. (2016). Inhibition of the processing of miR-25 by HIPK2-Phosphorylated-MeCP2 induces NOX4 in early diabetic nephropathy. Sci. Rep..

[68] Gawronski K.A.B., Kim J. (2017). Single cell transcriptomics of noncoding RNAs and their cell-specificity. Wiley Interdiscip. Rev. RNA.

[69] Flemming N., Pernoud L., Forbes J., Gallo L. (2022). Mitochondrial Dysfunction in Individuals with Diabetic Kidney Disease: A Systematic Review. Cells.

[70] Zhou X., Huang J., Zhang D., Qian Z., Zuo X., Sun Y. (2025). Small extracellular vesicles: The origins, current status, future prospects, and applications. Stem Cell Res. Ther..

[71] Wang W., Qiao S., Kong X., Zhang G., Cai Z. (2025). The role of exosomes in immunopathology and potential therapeutic implications. Cell. Mol. Immunol..

[72] Xie S., Zhang Q., Jiang L. (2022). Current Knowledge on Exosome Biogenesis, Cargo-Sorting Mechanism and Therapeutic Implications. Membranes.

[73] Liu J., Zhang Y., Tian Y., Huang W., Tong N., Fu X. (2022). Integrative biology of extracellular vesicles in diabetes mellitus and diabetic complications. Theranostics.

[74] Choezom D., Gross J.C. (2022). Neutral sphingomyelinase 2 controls exosome secretion by counteracting V-ATPase-mediated endosome acidification. J. Cell Sci..

[75] Zhou X., Wang L., Zou W., Chen X., Roizman B., Zhou G.G. (2020). hnRNPA2B1 Associated with Recruitment of RNA into Exosomes Plays a Key Role in Herpes Simplex Virus 1 Release from Infected Cells. J. Virol..

[76] Wang S., Shu J., Wang N., He Z. (2025). Exosomal non-coding RNAs: Mediators of crosstalk between cancer and cancer stem cells. Cell Death Discov..

[77] Krause M., Samoylenko A., Vainio S.J. (2015). Exosomes as renal inductive signals in health and disease, and their application as diagnostic markers and therapeutic agents. Front. Cell Dev. Biol..

[78] Zhao S., Li W., Yu W., Rao T., Li H., Ruan Y., Yuan R., Li C., Ning J., Li S. (2021). Exosomal miR-21 from tubular cells contributes to renal fibrosis by activating fibroblasts via targeting PTEN in obstructed kidneys. Theranostics.

[79] Ganesh V., He R., Martin J.A., Salem A.K., Sander E.A., Shin K., Seol D. (2025). Systematic review of extracellular vesicle-derived microRNAs involved in organ fibrosis: Implications for arthrofibrosis therapy. J. Transl. Med..

[80] Kato M., Natarajan R. (2015). MicroRNAs in diabetic nephropathy: Functions, biomarkers, and therapeutic targets. Ann. N. Y. Acad. Sci..

[81] Szostak J., Goracy A., Durys D., Dec P., Modrzejewski A., Pawlik A. (2023). The Role of MicroRNA in the Pathogenesis of Diabetic Nephropathy. Int. J. Mol. Sci..

[82] Wang B., Yao K., Huuskes B.M., Shen H.H., Zhuang J., Godson C., Brennan E.P., Wilkinson-Berka J.L., Wise A.F., Ricardo S.D. (2016). Mesenchymal Stem Cells Deliver Exogenous MicroRNA-let7c via Exosomes to Attenuate Renal Fibrosis. Mol. Ther..

[83] Chen T., Zhu J., Cai T., Du W., Zhang Y., Zhu Q., Liu Z., Huang J.A. (2019). Suppression of non-small cell lung cancer migration and invasion by hsa-miR-486-5p via the TGF-beta/SMAD2 signaling pathway. J. Cancer.

[84] Li Q., Zhang Z., Yin M., Cui C., Zhang Y., Wang Y., Liu F. (2022). What do we actually know about exosomal microRNAs in kidney diseases?. Front. Physiol..

[85] Sole C., Moline T., Vidal M., Ordi-Ros J., Cortes-Hernandez J. (2019). An Exosomal Urinary miRNA Signature for Early Diagnosis of Renal Fibrosis in Lupus Nephritis. Cells.

[86] Tello-Flores V.A., Valladares-Salgado A., Ramirez-Vargas M.A., Cruz M., Del-Moral-Hernandez O., Cahua-Pablo J.A., Ramirez M., Hernandez-Sotelo D., Armenta-Solis A., Flores-Alfaro E. (2020). Altered levels of MALAT1 and H19 derived from serum or serum exosomes associated with type-2 diabetes. Noncoding RNA Res..

[87] Liu X., Li M.H., Zhao Y.Y., Xie Y.L., Yu X., Chen Y.J., Li P., Zhang W.F., Zhu T.T. (2024). LncRNA H19 deficiency protects against the structural damage of glomerular endothelium in diabetic nephropathy via Akt/eNOS pathway. Arch. Physiol. Biochem..

[88] Kolling M., Kaucsar T., Schauerte C., Hubner A., Dettling A., Park J.K., Busch M., Wulff X., Meier M., Scherf K. (2017). Therapeutic miR-21 Silencing Ameliorates Diabetic Kidney Disease in Mice. Mol. Ther..

[89] Al-Kafaji G., Al-Mahroos G., Al-Muhtaresh H.A., Skrypnyk C., Sabry M.A., Ramadan A.R. (2016). Decreased expression of circulating microRNA-126 in patients with type 2 diabetic nephropathy: A potential blood-based biomarker. Exp. Ther. Med..

[90] Li S.Y., Susztak K. (2016). The long noncoding RNA Tug1 connects metabolic changes with kidney disease in podocytes. J. Clin. Investig..

[91] Fukuda A., Sato Y., Shibata H., Fujimoto S., Wiggins R.C. (2024). Urinary podocyte markers of disease activity, therapeutic efficacy, and long-term outcomes in acute and chronic kidney diseases. Clin. Exp. Nephrol..

[92] Bajan S., Hutvagner G. (2020). RNA-Based Therapeutics: From Antisense Oligonucleotides to miRNAs. Cells.

[93] Dhas Y., Arshad N., Biswas N., Jones L.D., Ashili S. (2023). MicroRNA-21 Silencing in Diabetic Nephropathy: Insights on Therapeutic Strategies. Biomedicines.

[94] Naeem S., Zhang J., Zhang Y., Wang Y. (2025). Nucleic acid therapeutics: Past, present, and future. Mol. Ther. Nucleic Acids.

[95] Chen H.Y., Zhong X., Huang X.R., Meng X.M., You Y., Chung A.C., Lan H.Y. (2014). MicroRNA-29b inhibits diabetic nephropathy in db/db mice. Mol. Ther..

[96] Tang Q., Khvorova A. (2024). RNAi-based drug design: Considerations and future directions. Nat. Rev. Drug Discov..

[97] Wilson B., Geetha K.M. (2022). Lipid nanoparticles in the development of mRNA vaccines for COVID-19. J. Drug Deliv. Sci. Technol..

[98] Li X., Li J., Wei J., Du W., Su C., Shen X., Zhao A., Xu M. (2025). Design Strategies for Novel Lipid Nanoparticle for mRNA Vaccine and Therapeutics: Current Understandings and Future Perspectives. MedComm.

[99] Wang Y., Wang N., Qu L., Jiao Y., Xiao Z. (2025). Nanomedicine for Acute Kidney Injury: Precision Delivery Strategies, Therapeutic Breakthroughs, Challenges, and Future Perspectives. Int. J. Nanomed..

